# Systemic shRNA mediated knock-down of S100A4 in colorectal cancer xenografted mice reduces metastasis formation

**DOI:** 10.18632/oncotarget.572

**Published:** 2012-08-07

**Authors:** Mathias Dahlmann, Ulrike Sack, Pia Herrmann, Margit Lemm, Iduna Fichtner, Peter M. Schlag, Ulrike Stein

**Affiliations:** ^1^ Experimental and Clinical Research Center, a joint cooperation between the Charité, Medical Faculty and the Max-Delbrück-Center for Molecular. Medicine, Berlin, Germany; ^2^ Max-Delbrück-Center for Molecular. Medicine, Germany; ^3^ Charité Comprehensive Cancer Center, Germany

**Keywords:** colorectal, cancer, metastasis, S100A4, RNAi

## Abstract

The metastasis-inducing protein S100A4 was found to be a prognostic indicator for the development of metachronous metastases. S100A4 expression levels correlate with the formation of human colorectal cancer metastases and shorter patients’ survival. Inhibition of S100A4 expression in patients might therefore result in decreased metastasis formation and prolonged survival. In the present study, we used shRNA expression plasmids to inhibit S100A4 expression in the colorectal cancer cell lines HCT116, SW620 and DLD-1. Cell lines with reduced S100A4 expression showed reduced cell migration and invasion *in vitro*. The knock-down of S100A4 expression also led to significantly diminished formation of liver metastases when intrasplenically transplanted in mice (*P* = 0.004). We then focused on the therapeutic potential of systemically applied shRNA expression plasmids acting on S100A4 via repeated hydrodynamics-based tail vein injection of plasmid DNA. Mice, intrasplenically transplanted with HCT116 cells and treated systemically with S100A4-shRNA plasmids, showed a decrease of S100A4 and MMP9 expression levels, resulting in significantly reduced liver metastases (*P* = 0.005). In summary, we show for the first time the intratumoral knock-down of S100A4 via systemic application of S100A4-shRNA plasmid DNA, which restricts metastasis formation in a xenografted mouse model of colorectal cancer.

## INTRODUCTION

Colorectal cancer is the third most common type of cancer worldwide, with more than 1.2 million new cases in the year 2008, and the second leading cause of cancer-related deaths in the Western world [1]. Although the 5-year-survival rate of patients diagnosed with colorectal cancer at an early stage is about 90%, it drops to 65% if regional lymph node metastases occur, and decreases to less than 10% if the patient is diagnosed with distant metastases [2].

The transformation of normal colon tissue to malignant cancer is caused by deregulated expression of both cancer promoting and inhibiting factors, often affecting tissue specific signaling pathways. Constitutively active Wnt/β-catenin signaling interferes with cell differentiation and promotes uncontrolled cell proliferation [3]. Consistently, almost all cases of colorectal cancer contain mutations in the Wnt/β-catenin signaling pathway, leading to sustained expression of its target genes [4].

One of the target genes of β-catenin regulated transcription is S100A4, also known as mts1 [5]. Overexpression of S100A4 in colorectal cancer tissue correlates with higher metastasis formation and is associated with a decrease of the patients’ survival rate [6],[7]. Although no increased tumor formation was found in S100A4 transgenic mice, metastasis formation was highly promoted, when crossed with mice that form tumors spontaneously. In turn, genomic knock-out of S100A4 strongly decreased the metastasis formation of highly metastasizing cancer cells in mice [8-10]. The ability of S100A4 to promote metastasis formation is mainly based on the variety of its interaction partners, which are involved in cellular migration, invasion, adhesion or angiogenesis [11-13]. Additionally, S100A4 can regulate the interplay of matrix metalloproteinases (MMPs) and their endogenous inhibitors (TIMPs), which are involved in invasion processes [14-16].

The inhibition of S100A4 expression or function can reduce the malignancy caused by enhanced S100A4 protein levels and, if used in clinical application, may increase the metastasis free survival of cancer patients. Several studies have reported strategies to decrease S100A4 expression in cell lines and tumor tissue. Some focused on the inhibition of the Wnt/β-catenin signaling activity with small molecules to reduce S100A4 at the transcriptional level [17-20]. Others focused on the posttranscriptional hydrolysis of S100A4 mRNA in cell lines, by transfection of catalytically active ribozymes or employing the RNA interference mechanism, with short interfering (si) RNA or the expression of short hairpin (sh) RNA directed against the S100A4 mRNA sequence [21-23].

In the present study, we used gene specific shRNA to inhibit S100A4 expression in the colorectal cancer cell lines HCT116, SW620, and DLD-1, resulting in reduced cell motility *in vitro* and, for HCT116, reduced metastasis formation after xenograft transplantation *in vivo*. For the first time, we performed systemic, hydrodynamics-based delivery of S100A4-shRNA expression plasmids thereby decreasing the S100A4 expression in the tumor tissue of xenografted mice for colorectal cancer, which led to reduced metastasis formation *in vivo*.

## RESULTS

### Down-regulation of S100A4 by stable, plasmid based shRNA expression inhibits cellular migration and invasion *in vitro*

**Figure 1:**
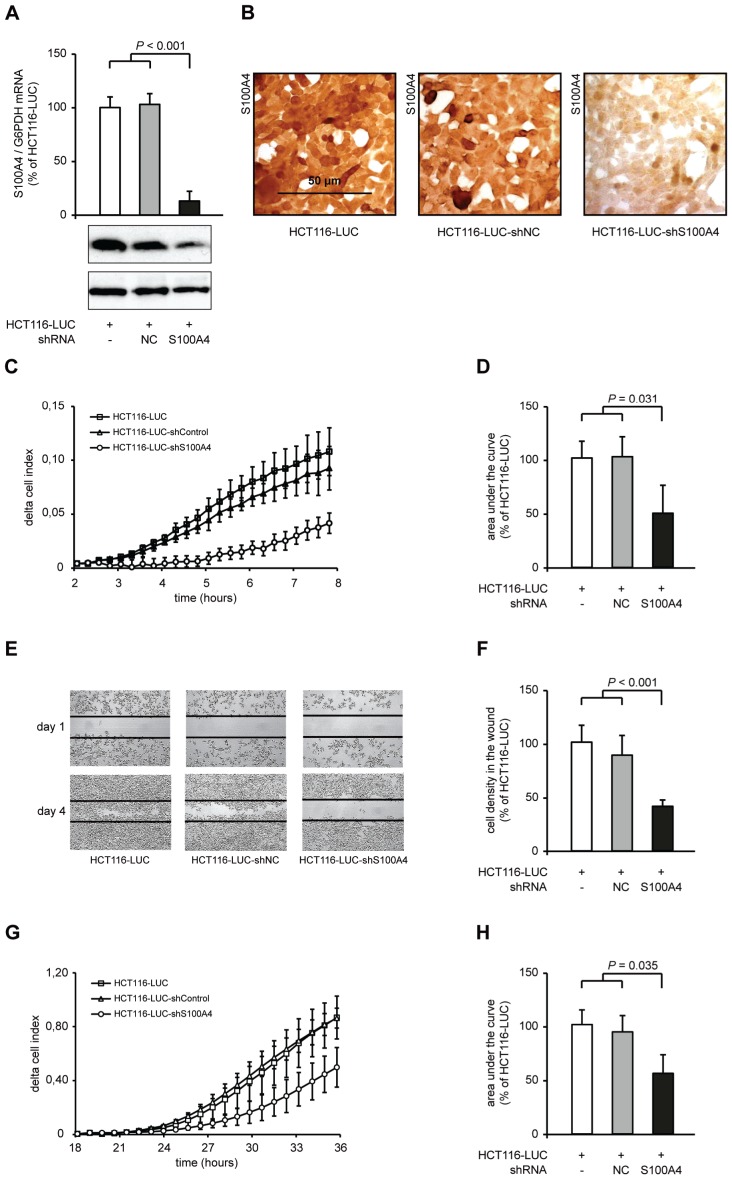
S100A4-shRNA reduces S100A4 expression and cellular motility in HCT116 (A) qRT-PCR analysis of S100A4 mRNA and Western blot analysis of S100A4 protein level of HCT116-LUC, HCT116-LUC-shNC, and HCT116-LUC-shS100A4 cells. Data represent mean S100A4 / G6PDH mRNA ratios (n = 4) ± SD. Results of HCT116-LUC cells were set to 100%. Equal loading at Western blot analysis of S100A4 protein in the cell lines was confirmed by GAPDH protein detection. (B) Immunocytochemistry staining of HCT116-LUC, HCT116-LUC-shNC, and HCT116-LUC-shS100A4 cells using anti-S100A4 antibody. Nuclei were stained with hematoxylin. Scale bar represents 50 μm. (C) Real time monitoring of cellular migration with the xCELLigence DP system. The signals of HCT116-LUC, HCT116-LUC-shNC and HCT116-LUC-shS100A4 cells were normalized at 2 hours. Data indicate mean (n = 3) ± SD. (D) Differences in cell index, due to cell migration over time, were quantified by integrating the normalized curves between 2 hours and 8 hours. Results of HCT116-LUC-shNC and HCT116-LUC-shS100A4 cells were normalized to HCT116-LUC cells. Data represent mean (n = 3) ± SD. (E) Wound healing assay for directed migration of HCT116-LUC, HCT116-LUC-shNC and HCT116-LUC-shS100A4 cells. A 0.3 mm scratch was set in a semi confluent cell layer and cells were monitored until day 4. Representative images were taken to illustrate the closing of the wound. (F) Quantification of migrated HCT116-LUC, HCT116-LUC-shNC and HCT116-LUC-shS100A4 cells, by image analysis of the scratch. Data represent mean densities in the cell layer wound (n = 3) ± SD. (G) Real time monitoring of cell invasion. The signals of the cell lines HCT116-LUC, HCT116-LUC-shNC and HCT116-LUC-shS100A4 were normalized at 12 hours. Data represent mean (n = 3) ± SD. (H) Differences in cell index, due to Matrigel invasion over time, were quantified by calculating the area under the normalized curves between 12 hours and 36 hours. Results of HCT116-LUC-shNC and HCT116-LUC-shS100A4 cells were normalized to HCT116-LUC cells. Data represent mean (n = 3) ± SD.

We began our S100A4 knock down studies using HCT116-LUC, HCT116-LUC-shNC and HCT116-LUC-shS100A4 cells. HCT116-LUC cells also express firefly luciferase. HCT116-LUC-shNC and HCT116-LUC-shS100A4 cells were additionally transfected with either an unrelated control shRNA-sequence (negative control, NC) or an S100A4 specific shRNA. S100A4 mRNA level showed a significant reduction to 13% in HCT116-LUC-shS100A4 cells, compared to HCT116-LUC and HCT116-LUC-shNC cells, respectively (*P <* 0.001). Western blotting of total cell lysates and immunostaining against S100A4 confirmed the decrease of endogenous S100A4 expression level in S100A4-shRNA transfected cells, compared to the respective control cells (Figure 1A). Immunocytochemistry of HCT116-LUC, HCT116-LUC-shNC and HCT116-LUC-shS100A4 cells demonstrated a high expression of S100A4 in HCT116-LUC and HCT116-LUC-shNC cells, but a strong S100A4 protein reduction in HCT116-LUC-shS100A4 cells (Figure 1B).

S100A4 has previously been linked to enhanced tumor migration and formation of metastasis of colorectal cancer [6],[24],[25]. Therefore, we analyzed the ability of these cells to migrate through porous membranes with the xCELLigence system, which allows real time data recording of cellular processes. In the xCELLigence-based assay, migrated cells attach on the bottom side of the membrane and increase the electrical impedance at the electrodes. HCT116-LUC-shS100A4 cells showed a delay of the signal increase of almost 3 hours and a lower signal increase than the control cells HCT116-LUC and HCT116-LUC-shNC (Figure 1C). We integrated the area under the signal curves of independent experiments and observed a significant reduction of migrating HCT116-LUC-shS100A4 cells to 49% (*P* = 0.031) compared to the control cell lines HCT116-LUC and HCT116-LUC-shNC (Figure 1D).

The directed cellular migration was evaluated by closing an applied scratch in a cell layer, documented daily until day 4. HCT116-LUC-shS100A4 cells showed a strong delay in wound closure compared to the control cell lines (Figure 1E). The closure of the wound was quantified by image analysis, resulting in a decrease of 41% in HCT116-LUC-shS100A4 cells (*P* < 0.001), compared to the control cell lines HCT116-LUC and HCT116-LUC-shNC (Figure 1F).

Beside increased migration, cancer cells have to pass through an intercellular matrix barrier to invade adjacent tissues and form distant metastases. We measured the ability of the cell lines to penetrate an extracellular matrix (ECM) like structure by adding a layer of Matrigel on top of the membranes. Using the xCELLigence system, S100A4-shRNA transfected cells showed a lower increase of the cell index after 24 hours (Figure 1G). The integration of the curves showed a decrease to 55% (*P* = 0.035), compared to the control cells (Figure 1H). The xCELLigence-based *in vitro* motility assays were confirmed by classical Boyden chamber assays for cell migration and invasion (Figure S1A,B).

We also analyzed the proliferative abilities of HCT116-LUC, HCT116-LUC-shNC, and HCT116-LUC-shS100A4 cells. However, neither the doubling time nor the ability to form colonies in soft agar differed significantly (Figure S1C,D).

**Figure 2:**
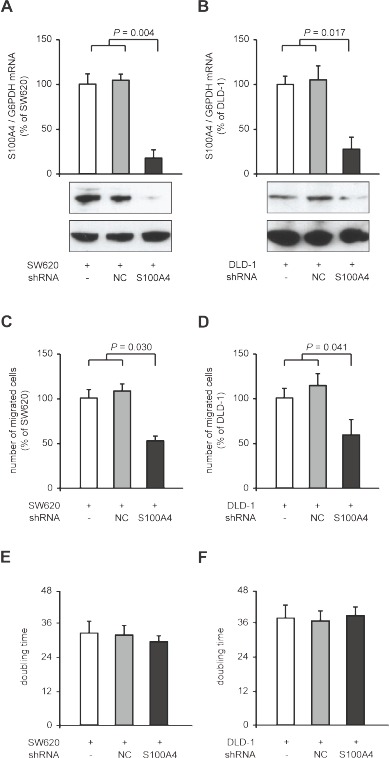
Reduction of cellular migration in SW620 and DLD-1 colorectal cancer cell lines after S100A4-shRNA transfection qRT-PCR analysis of S100A4 mRNA and Western blot analysis of protein level in parental SW620, SW620-shNC and SW620-shS100A4 cells (A), and in parental DLD-1, DLD-1-shNC and DLD-1-shS100A4 cells (B), respectively. Data represent mean S100A4 / G6PDH mRNA ratios (n = 4) ± SD. Results of parental cells were set to 100%. Equal loading at Western blot analysis of S100A4 protein in the cell lines was confirmed by GAPDH protein detection. (C,D) Boyden Chamber assay of cell migration. Data represent the number of migrated parental and shRNA transfected cell lines derived from SW620 and DLD-1, normalized to the parental SW620 and DLD-1 cell lines, respectively (as mean (n = 3) ± SD). (E,F) Doubling times of parental and shRNA transfected cell lines derived from SW620 and DLD-1, respectively, were determined by MTT assays. Data represent mean values (n = 3) ± SD.

We verified the reduction of cellular motility after S100A4 knock-down in the colorectal cancer cell lines SW620 and DLD-1. We generated stably shRNA transfected clones thereof, SW620-shNC and SW620-shS100A4, as well as DLD-1-shNC and DLD1-shS100A4. In SW620-shS100A4 and DLD-1-shS100A4 cells, S100A4 mRNA levels were reduced to 17% (*P* = 0.004) and 28% (*P* = 0.017), respectively, compared to the respective control cell lines containing either no or control shRNA (Figure 2A,B). By counting migrated cells in the Boyden chamber assay, we observed a reduction in cell migration in the cell lines SW620-shS100A4 to 53% (*P* = 0.030), and DLD-1-shS100A4 to 59% (*P* = 0.041), compared to the respective control cells (Figure 2C,D). No change in the doubling time of the cell lines derived from SW620 and DLD-1 was observed (Figure 2E,F).

### Transplantation of S100A4 knock-down cells reduces metastasis formation in mice

**Figure 3:**
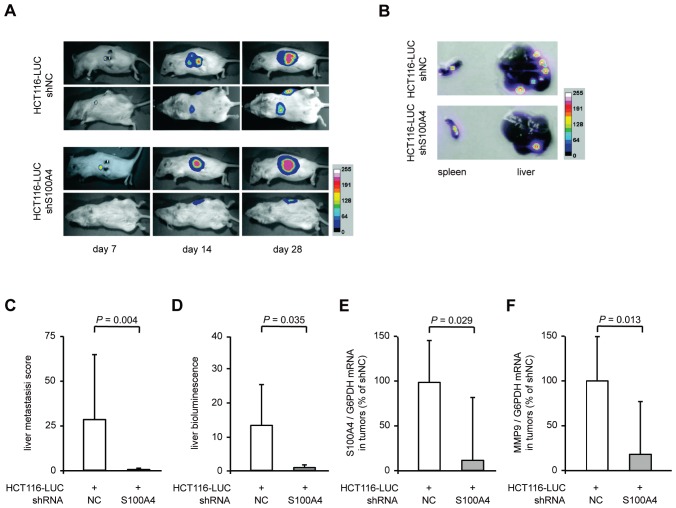
Stable S100A4 knock down in transplanted HCT116-LUC cells reduces metastasis formation *in vivo* The cell lines HCT116-LUC-shNC and HCT116-LUC-shS100A4 were transplanted into the spleen of NOD/SCID mice (n = 8 per group). (A) *In vivo* images of a representative animal of each group in lateral and dorsal position, respectively, depicted at days 7, 14, and 28 after transplantation. Luminescence and bright field images were combined as an overlay picture. The color bar indicates the signal strength after 5 minutes exposure time. (B) After sacrificing, spleens and livers were removed of each animal and *ex vivo* imaging was performed separately. The spleens and livers depicted originate from the representative animals shown in (A). The color bar indicates the signal strength after 20 seconds exposure time. (C) The level of metastasis was evaluated by scoring. The score for each liver was calculated as the sum of the volumes of the individual metastases. Data represent the mean liver metastasis score (n = 8) ± SD of each group. (D) Quantification of *ex vivo* bioluminescence intensities of isolated livers. Data represent mean intensities (n = 8) ± SD of each group. (E,F) Ratios of S100A4 / G6PDH mRNA and MMP9 / GAPDH mRNA in primary tumor tissues were determined by qRT-PCR. Data represent mean (n = 8) ± SD. Results of transplanted HCT116-LUC-shNC cells were set to 100%.

Our results showed a clear decrease in the metastatic potential of colorectal cancer cells, when stably transfected with S100A4-shRNA expression plasmids. To evaluate the anti-metastatic effect of S100A4 knock-down *in vivo*, the cell lines HCT116-LUC-shNC and HCT116-LUC-shS100A4 were transplanted into the spleens of female NOD/SCID mice (n = 8, each group). The ability of the cells to form metastases in the liver was examined by *in vivo* bioluminescence imaging of animals and *ex vivo* scoring of visible metastases after organ resection (Figure 3). Both groups showed luminescence signals near the transplantation site, which increased over time during the experiment (Figure 3A, rows 1 and 3). However, additional luminescence signals distant from the transplantation site were only observed in the control mice (Figure 3A, rows 2 and 4). After the sacrifice of the animals, spleens and livers were removed. The tumors in the spleens of representative mice and the metastatic burden of the corresponding livers are shown as luminescence overlays (Figure 3B). Scoring of the liver metastases showed a significant decrease of number and size of metastases in the animals with transplanted HCT116-LUC-shS100A4 cells (*P* = 0.004). Liver metastases of control mice had an average score of 27.3 (± 35.1), whereas liver metastases of the mice with transplanted HCT116-LUC-shS100A4 cells had an average score of 0.3 (± 0.8) (Figure 3C). Quantification of bioluminescence signal intensities of organs confirmed the reduction of liver metastases with transplanted HCT116-LUC-shS100A4 cells (*P* = 0.035) compared to transplanted HCT116-LUC-shNC cells (Figure 3D). No significant differences were found in primary tumor size, average body weight, as well as the weight of spleens and livers (Table 1). The reduction of S100A4 expression in tumor tissue of transplanted HCT116-LUC-shS100A4 mice was verified by qRT-PCR (Figure 3E). When we analyzed changes in the expression of genes associated with S100A4-mediated metastasis formation, we observed a significant decrease of matrix metalloproteinase (MMP) 9 in the tumor tissue derived from HCT116-LUC-shS100A4 cells (Figure 3F).

**Table 1:** Comparison of body weights, organ weights and bioluminescence signal intensities of spleens (tumors) and livers (metastases) of xenografted mice

Transplanted cell line ^1^		Body weight (gram) mean ± SD	Spleen weight (gram) mean ± SD	Liver weight (gram) mean ± SD	Bioluminescence signal intensity mean ± SD	
					Spleen	Liver
HCT116-LUC-shNC (n = 8) HCT116-LUC-shS100A4 (n = 8)		20.8 ± 1.6 19.3 ± 0.9	0.05 ± 0.01 0.06 ± 0.03	1.2 ± 0.1 1.1 ± 0.1	18.1 ± 11.3 15.7 ± 12.1	13.1 ± 11.9 0.8 ± 0.8
		n.s.	n.s.	n.s.	n.s.	*P* = 0.035
**Transplanted cell line ^1^**	**Plasmid (t.v.i.) ^2^**					
HCT116-LUC	p-shNC (n = 8) p-shS100A4 (n = 8)	18.5 ± 2.0 18.4 ± 1.9	0.18 ± 0.07 0.20 ± 0.07	1.2 ± 0.1 1.1 ± 0.2	16,4 ± 8,4 14,1 ± 8,2	21,9 ± 20,8 4,5 ± 4,3
		n.s.	n.s.	n.s.	n.s.	*P* = 0.032

Abbreviations: SD, standard deviation; LUC, firefly luciferase; shNC, negative control shRNA; shS100A4, S100A4 specific shRNA; t.v.i., tail vein injection

1 xenografted mice with intrasplenic transplantation of indicated cell lines;

2 continued systemic application of shRNA expression plasmids, via tail vein injection;

data represent mean ± SD; statistical analysis of groups was done by Mann-Whitney-Rank-Sum-Test.

### Systemic application of S100A4-shRNA expression plasmids reduced metastasis formation in mice

**Figure 4:**
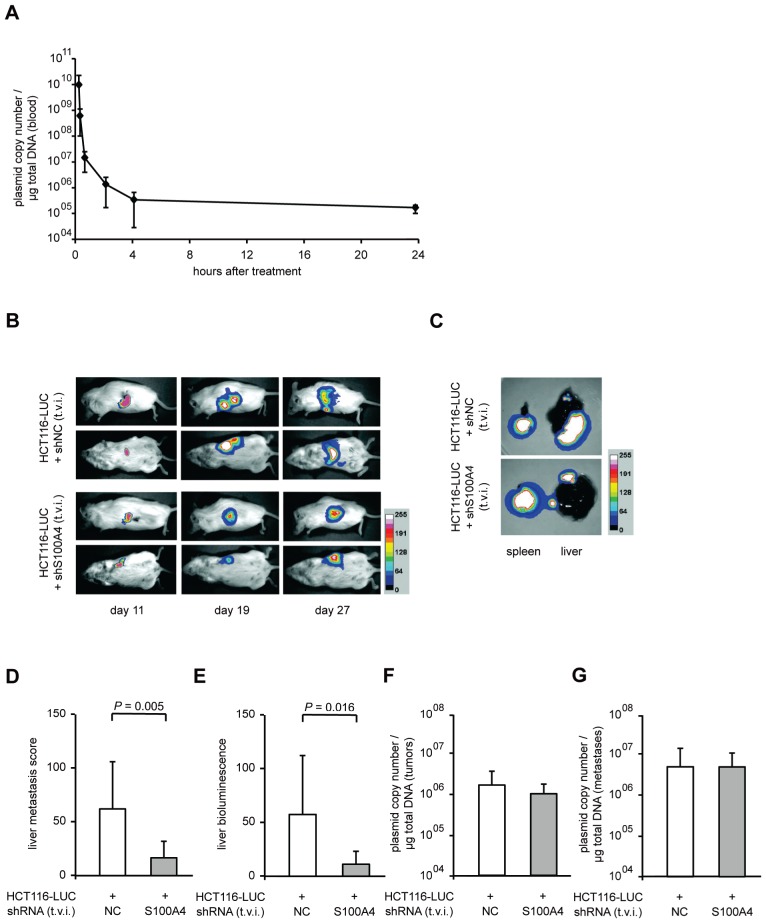
Systemic application of S100A4-shRNA-plasmids reduced metastasis formation of intrasplenically transplanted HCT116-LUC cells *in vivo* (A) Plasmid concentration in blood after single tail vein injection. Blood samples were taken before and 5 minutes, 30 minutes, and 1, 2, 4 and 24 hours after a single tail vein injection (t.v.i) of ten μg shRNA-expression plasmid, supplemented with 2 μg aurintricarbolic acid (ATA), in 200 μl PBS into NOD/SCID mice. Plasmid copy numbers per μg of total DNA were determined by qPCR. Data represent mean (n = 5) ± SD. Randomly selected groups of NOD/SCID mice with intrasplenically transplanted HCT116-LUC cells (n = 8 per group) were treated with systemically applied shRNA-plasmids (negative control and shS100A4, respectively) via repeated tail vein injection. (B) *In vivo* images of representative mice of each group at lateral and dorsal position, depicted at days 11, 19, and 27 after transplantation. Luminescence and bright field images were combined as overlay pictures. The color bar indicates the signal strength after 5 minutes exposure time. (C) After sacrificing, spleens and livers were removed of each animal and *ex vivo* imaging was performed separately. The spleens and livers depicted originate from the representative animals shown in (B). The color bar indicates the signal strength after 20 seconds exposing time. (D) The level of metastasis was evaluated by scoring. The score for each liver was calculated as the sum of the volumes of the individual metastasis. Data represent the mean liver metastasis score (n = 8) ± SD of each group. (E) Quantification of *ex vivo* bioluminescence intensities of isolated livers. Data represent mean intensities (n = 8) ± SD of each group. (F,G) Copy number of each shRNA expression plasmid per μg of total DNA, isolated from spleen and liver (respectively) of each animal and quantified via amplification of a plasmid backbone specific amplicon. Data represent mean (n=8) ± SD, of each group.

In order to evaluate therapeutic approaches to interfere with S100A4 driven metastasis formation, we improved the experimental setup by systemic application of S100A4 shRNA plasmids. First, we determined the plasmid copy number of systemically administered shRNA expression plasmids (10 μg plasmid DNA) in the blood of NOD/SCID mice (n = 5) over time before and 5 minutes, 30 minutes, as well as 1, 2, 4, and 24 hours after single tail vein injection. Although the plasmid is cleared rapidly in the blood, 10^5^ copies per μg of total DNA were detectable after 24 hours (Figure 4A).

Based on this, the cell line HCT116-LUC was transplanted intrasplenically into female NOD/SCID mice, and the animals were randomly divided into two groups (n = 8, each). We then applied shRNA expression plasmids systemically by repeated tail vein injection every second day until day 26. One group was treated with shRNA plasmids specific for S100A4, the other with a plasmid containing an unrelated control sequence. As we have observed before, the *in vivo* luminescence signals at the site of transplantation increased over time in both groups (Figure 4B, rows 1 and 3). However, an increase of distant, additional signals was mainly observed in control mice (Figure 4B, rows 2 and 4). The tumors in the spleens of representative mice and the metastatic burden of the corresponding livers are shown as *ex vivo* luminescence overlays (Figure 4C). The average score of liver metastases in shS100A4 treated animals was also statistically lower, compared to control mice, 16.0 (± 14.8) and 59.9 (± 42.6), respectively (*P* = 0.005, Figure 4D). Consistent with our previous data, the quantification of bioluminescence signal intensities of livers and spleens confirmed the reduction in liver metastases in animals treated with S100A4 shRNA expression plasmids (*P* = 0.016), compared to a treatment with plasmids for control shRNA expression (Figure 4E). No significant difference in primary tumor size, average body weight or the weight of spleens and livers were detectable (Table 1). By plasmid specific qPCR, we quantified the copy number of each plasmid and determined a similar plasmid concentration of about 1 × 10^6^ copies per μg of total DNA in primary tumors and 4 × 10^6^ copies per μg of total DNA in liver metastases, respectively, in both animal groups (Figure 4F,G).

**Figure 5:**
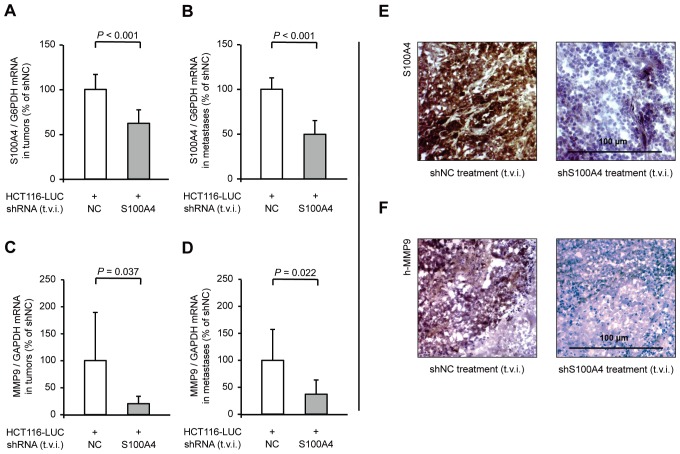
Tail vein injection of S100A4-shRNA-plasmids reduces S100A4 and MMP9 expression in tumors and metastases Ratios of S100A4 mRNA and MMP9 mRNA to house-keeping gene expression were determined by qRT-PCR. Data represent mean (n = 8) ± SD. Results of treatment with shNC expression plasmids were set to 100%. (A) S100A4 / G6PDH mRNA expression of primary tumors; (B) S100A4 / G6PDH mRNA expression of liver metastases; (C) MMP9 / GAPDH mRNA expression of primary tumors; (D) MMP9 / GAPDH mRNA expression of liver metastases. Immunhistochemistry staining of representative spleen tumor sections of each group for S100A4 (E) and MMP9 (F), respectively. Nuclei were stained with hematoxylin. The scale bar represents 100 μm.

Subsequent analysis of relative S100A4 mRNA levels in micro-dissected spleen and liver tissue showed a significant decrease (*P* < 0.001) in those animals, treated with shS100A4 expression plasmids, to 62% in tumors and 50% in metastases of the control mice (Figure 5A,B). Again, we found the expression level of MMP9 significantly reduced in mice with specific S100A4-shRNA treatment. MMP9 mRNA expression dropped to 20% (*P* = 0.037) in spleen tumor tissue and to 37% (*P* = 0.022) in liver metastases, compared to control mice (Figure 5C,D). Reduced S100A4 and MMP9 expression was confirmed on protein level by immunohistochemistry of tumor tissue from both groups (Figure 5E,F).

## DISCUSSION

In this study, we demonstrate the reduction of S100A4 induced *in vivo* metastasis formation in xenografted colorectal cancer tumors by systemic application of plasmids expressing S100A4-specific shRNA, via tail vein injection. The reduced S100A4 expression resulted in reduced expression of MMP9, leading to significantly decreased metastasis formation in treated mice.

Administration of naked DNA is a well-established tool for gene expression studies in animals as well as for gene therapy in clinical trials [26],[27]. Systemic application of small inhibiting RNAs, both siRNA and shRNA, via tail vein injection leads to an efficient, but transient decrease of target gene expression [28], and prolonged silencing of target genes has been achieved by using shRNA expression plasmids [29],[30]. To our knowledge, this is the first study that uses systemic application of plasmid based S100A4-shRNA expression to inhibit metastasis formation of colorectal cancer.

Growth, signaling, differentiation, and motility are susceptible to changes in S100A4 expression [11],[31] and the knock-down of S100A4 by RNA interference has been reported to inhibit cell growth and motility of various cancer cell lines like gastric cancer or osteosarcoma [32],[33]. In our study, the stable knock-down of S100A4 in the colorectal cancer cell lines HCT116, SW620, and DLD-1, by plasmid based shRNA expression, did not result in reduced cellular growth *in vitro*. This is consistent with studies of other groups, that also report no effect of S100A4 reduction on cellular growth [21],[22],[34]. Furthermore, by using the recently developed impedance based real-time cell analysis, we confirmed a decreased migration rate and a decreased invasion rate caused by knocked-down S100A4 expression in HCT116 cells. In addition, we verified these effects in the colorectal cancer cell lines SW620 and DLD-1 by classical Boyden chamber assays.

The reduction of cell motility *in vitro* also suggests a decrease in the metastatic potential of S100A4 knock-down cells in xenografted mice. We transplanted the HCT116-LUC-shNC cells into the spleens of NOD/SCID mice, leading to liver metastases [35]. In contrast, transplantation of HCT116-LUC-shS100A4 cells with stable down regulation of S100A4 expression resulted in significantly less liver metastases in mice. This effect has also been shown for RNA interference of S100A4 expression in osteosarcoma and esophageal squamous cell carcinoma, by using stable transfection or siRNA treatment previous of cell transplantation, respectively [34].

*In vivo* data of reduced S100A4 expression in colon cancer of other studies were mainly obtained by inhibiting the hyperactivated Wnt/β-catenin pathway and therefore indirectly reducing the S100A4 transcription [5],[17],[19],[20]. Only one S100A4 protein-specific peptide inhibitor has been reported so far, disrupting the molecular interaction of S100A4 with Myosin-IIA [36]. Therefore, we focused on the knock-down of S100A4 expression by systemic application of plasmids for expression of S100A4 specific shRNA.

It was previously shown, that hydrodynamics-based transfected plasmid DNA in mice is predominantly delivered to the liver, but also to spleen, kidney, lung, and heart [37]. Moreover, the stable knock-down of gene expression by systemic application of shRNA expression plasmids seems to be 15 to 250 times more efficient than transient knock- down approaches with siRNA [38],[39]. In our study, we observed a significant decrease of liver metastases to less than 30% of the control group by systemic application of S100A4-shRNA expression plasmids. This was caused by reduced S100A4 mRNA expression in tumors and metastases of human colorectal cancer xenografted mice. It also resulted in decreased MMP9 mRNA expression in tumors and in metastases. The reduction of S100A4 and MMP9 was confirmed by immunohistochemistry of tumor tissue samples. The role of S100A4 in regulating matrix metalloprotease expression and activity has been previously reported [14],[40]. In the case of MMP9, several studies in different types of cancer report either an increase in MMP9 activity with elevated S100A4 expression levels or a decrease of MMP9 expression with a knock-down of S100A4 [15],[41-44] One possible mechanism, which is discussed for the S100A4 dependent MMP9 regulation, is the direct or indirect activation of p53 or JNK, since S100A4 is a binding partner of p53 and can modulate its activity [45],[46]. An additional mechanism may include the interaction of S100A4 with Smad3, a member of the TGF-β/Smad pathway, which also regulates MMP9 expression [44],[47]. The complexity of protein interactions and regulated pathways might be dependent on the tumor entity and requires further investigations to understand tissue specific metastasis promoting factors.

Since 40% of colorectal cancer patients form distant metastases after initial resection of the primary tumor, the development of adjuvant anti-metastatic treatment options is needed [48]. Here we demonstrate for the first time that the systemic application of plasmid DNA for S100A4-specific shRNA decreased S100A4 and MMP9 expression levels in tumors and metastases, and, most importantly, reduced formation of liver metastasis. Thus, a personalized RNA interference approach to reduce the S100A4 expression in those patients with high S100A4 expressing primary tumors might serve as an alternative or addition to adjuvant chemotherapeutic protocols for prevention of metastasis formation in colorectal cancer.

## METHODS

### Ethics statement

Investigation has been conducted in accordance with the ethical standards and according to the Declaration of Helsinki and according to national and international guidelines and has been approved by the authors’ institutional review board.

### Cell culture and transfection

The human colorectal cancer cell line HCT116 was stably transfected with a pcDNA3.1 plasmid, carrying firefly luciferase under the CMV promoter, and the puromycin resistance cassette [19]. The resulting cell line HCT116-LUC was further transfected with SureSilencing™ control or S100A4 shRNA expression plasmids, containing the neomycin resistance cassette (SABiosciences, Frederick, MD). HCT116-LUC-shS100A4 cells express a shRNA, specifically targeting S100A4-mRNA, while HCT116-LUC-shNC cells (negative control) express an unrelated control sequence. Transfection of the cell lines SW620 and DLD-1 with the shRNA-expression plasmids resulted in the cell lines SW620-shS100A4, SW620-shNC, DLD-1-shS100A4, and DLD-1-shNC, respectively. All transfections were performed with Fugene HD (Roche Diagnostics, Germany), according to the manufacturer's instructions. Transfected cells were selected with 1 mg/ml neomycin (PAA Laboratories, Austria) or 1 μg/ml puromycin (Life Technologies Corporation, Carlsbad, CA). All cell lines derived from HCT116 and SW620 were grown in RPMI-1640 medium, cell lines derived from DLD-1 were grown in DMEM (PAA Laboratories), each medium supplemented with 10% FCS (Life Technologies Corporation), in a humidified incubator at 37°C and 5% CO_2_. The cell lines were tested with the MycoAlert® Mycoplasma Detection Kit (Lonza, Rockland, ME), and found to be free of mycoplasma. The genotype of the parental cell lines HCT116, SW620, and DLD-1 were confirmed by short tandem repeat (STR) genotyping at the DSMZ (German Collection of Microorganisms and Cell Cultures; Braunschweig, Germany). The STR genotypes were consistent with published genotypes for each cell line.

### Real-time Quantitative Reverse Transcriptase Polymerase Chain Reaction (qRT-PCR)

Total RNA from cell culture or tissues was isolated using the Universal RNA Purification Kit (Roboklon, Germany), according to the manufacturer's instructions. Quantification of RNA concentration was performed with Nanodrop (Peqlab, Germany), and 50 ng total RNA was reverse transcribed with random hexamers in a reaction mix (10 mM MgCl_2_, 1 × PCR-buffer II, 250 μM pooled dNTPs, 1 U/μL RNAse inhibitor, 2.5 U/μL MuLV reverse transcriptase; all from Life Technologies Corporation). Reaction occurred at 42°C for 15 minutes, 95°C for 5 minutes, and subsequent cooling at 4°C for 5 minutes. The cDNA product was amplified in a total volume of 10 μl in 96-well plates using the LightCycler 480 (Roche Diagnostics, Mannheim, Germany) and the following PCR conditions: 95°C for 10 minutes, followed by 45 cycles of 95°C for 10 seconds, 60°C for 30 seconds, and 72°C for 4 seconds. For S100A4 cDNA quantification, the following primer and probes were used for a 124 bp amplicon: forward primer, 5′-gagctgcccagcttcttg-3′; reverse primer, 5´-tgcaggacaggaagacacag-3´; fluorescein isothiocyanate (FITC) probe, 5′-tgatgagcaacttggacagcaaca-3′; and LCRed640-probe, 5′-gacaacgaggtggacttccaagagt-3′. For cDNA quantification of the housekeeping gene glucose-6-phosphate dehydrogenase (G6PDH) the LightCycler-h-G6PDH Housekeeping Gene Set (Roche Diagnostics) was used, according to manufacturer's instructions, leading to a 113 bp amplicon. For MMP9 and glyceraldehyde-3-phosphate dehydrogenase (GAPDH) cDNA quantification with a SYBR® Green based reaction mix (GoTaq®; Promega, Germany), the following primers were used: MMP9, forward-primer 5´-tggggggcaactcggc-3´, reverse-primer 5´-ggaatgatctaagcccag-3´, 224 bp amplicon; GAPDH, forward-primer 5´-gaaggtgaaggtcggagtc-3´, reverse-primer 5´-ggtggaatcatattggaacatgtaa-3´, 151 bp amplicon. Data analysis was performed with LightCycler 480 Software release 1.5.0 SP3 (Roche Diagnostics). Mean values were calculated from duplicate qRT-PCR reactions. Each mean value of the expressed gene was normalized to the respective mean value of the housekeeping gene cDNA. The cDNA for the calibrator and the in-run standard derived from HCT116 cells was employed in serial dilutions simultaneously in each run.

### Protein extraction and immunoblot

For total protein extraction, cells were lysed with RIPA buffer (50 mM Tris-HCl, 150 mM NaCl, 1% Nonidet P-40, supplemented with complete protease inhibitor tablets; Roche Diagnostics) for 30 minutes on ice. Protein concentration was quantified with Coomassie Plus (Bradford) Protein Assay Reagent (Pierce; Rockford, IL), according to manufacturer's instructions, and lysates of equal protein concentration were separated with SDS-PAGE and transferred to Hybond-C Extra nitrocellulose membrane (GE Healthcare; Germany). Membranes were incubated in blocking solution containing 5% nonfat dry milk for 1 hour at room temperature. Membranes were incubated overnight at 4°C with rabbit anti-human S100A4 antibody (Dako, Denmark; dilution 1:1000) or goat anti-human GAPDH antibody (Santa Cruz Biotechnology; dilution 1:500) followed by incubation for 1 hour at room temperature with HRP-conjugated anti-rabbit IgG (dilution 1:10000) or anti-goat IgG (dilution 1:10000), respectively. Antibody-protein-complexes were visualized with electrochemical-luminescence reagent (100 mM Tris/HCl, 0.025% w/v luminol, 0.011% w/v para-hydroxycoumaric acid, 10% v/v dimethylsulfoxide, 0.004% v/v H_2_O_2_, pH 8.6) and subsequent exposure to CL-XPosure™ Films (Pierce). Immunoblotting for GAPDH served as protein loading control. All experiments were performed at least three independent times.

### Immunocytochemistry and immunohistochemistry

For immunocytochemistry, cells were cultured on double-chamber slides (Nunc, Rochester, NY). For immunohistochemistry, cryosections (5 μm) of tumor tissue were transferred onto glass slides. Samples were fixated for 15 minutes with 0.04% glutaraldehyde, endogenous peroxidase was inactivated for 20 minutes with 0.9% H_2_O_2_, and cells were permeabilized for 10 minutes with 0.5% Triton X100 in 2.5% BSA. After blocking for 1 hour with 5% BSA, cells were incubated with the S100A4 antibody (dilution 1:400, 2 hours) or rabbit h-MMP9 antibody (Cell Signaling, dilution 1:400, 2 hours). Detection was performed using the biotin-based ABC kit (Dako, Denmark) and diaminobenzidine as substrate, according to manufacturer's instructions. Positive signals resulted in brown staining. The slides were counterstained with haematoxylin and analyzed using a light microscope Axioplan2 (Leica, Germany) at a magnification of 40x. Negative control experiments were carried out by omitting the primary antibody.

### Real-time electrical impedance–based monitoring of cell migration, invasion and proliferation

For monitoring of cell migration and invasion in real-time the xCELLigence Real Time Cell Analyzer Dual Plate (RTCA-DP) instrument was used according to the manufacturer's recommendations (Roche Applied Science, Germany). The impedance is expressed as a dimensionless parameter, termed cell index, and is directly proportional to the area covered by cells. For detection of cellular migration and invasion, electrical impedance changes are measured at a gold microelectrode plated on the bottom of a membrane separating the upper and lower chambers. The cell line (HCT116-LUC) and the clones expressing control- or S100A4-shRNA (HCT116-LUC-shNC and HCT116-LUC-shS100A4, respectively) were subjected to serum starvation, 12 hours before the start of measurement. For cell migration assays, 5 × 10^4^ cells in RPMI-1640 were seeded per well of a 16-well CIM plate, and the lower chamber was loaded with RPMI-1640, supplemented with 10% FCS. For cell invasion assays, the membranes were coated with Matrigel (dilution 1:40 with medium) 12 hours before seeding 1 × 10^5^ cells. Cell proliferation was determined by seeding 5 × 10^3^ cells per well into E-Plates and recording the cell index every 15 minutes for 75 hours. Quantitative measurements of changes of the electrical impedance are expressed as cell index. For cell migration, cell index values were monitored every 5 minutes for 24 hours. For invasion, the cell index was measured every 15 minutes for 48 hours. At least three independent experiments were performed for monitoring cell migration or invasion, respectively, each carried out in triplicates. Cell index values were calculated and plotted using the RTCA software 1.2.1 of the RTCA xCELLigence system. For cell migration, the cell index curves were normalized 2 hours after seeding and signals between 2 hours and 8 hours were used to calculate the area under the curve. For cell invasion, the cell index curves were normalized 12 hours after seeding and signals between 12 hours and 36 hours were used to calculate the area under the curves. Average values were normalized to HCT116-LUC.

### Wound healing assay

Wound healing assay was performed as described previously [19]. HCT116-LUC, HCT116-LUC-shNC and HCT116-LUC-shS100A4 cells were grown to form 60% confluent monolayers, in which a wound of about 0.3 mm width was set with a sterile pipette tip. The medium was exchanged to remove non-adherent cells. The progress of wound closure was monitored daily with microphotographs of 10 × magnification taken with the Leica DM IL light microscope and a Kappa CF 15/4 MCC-MLUII Modul camera (Leica Microsystems) up to day 4. The wound healing experiment was performed three independent times. The spreading of cells across the area of the wound at day 4 was quantified with ImageJ [49]. The background value (day 1) was substracted and the average values were normalized to HCT116-LUC.

### Monitoring cell proliferation by MTT

Two × 10^3^ cells of the cell lines SW620 and DLD-1, including the stable shRNA expression clones SW620-shNC, SW620-shS100A4, DLD-1-shNC and DLD-1-shS100A4, respectively, were seeded in 96-well-plates (for each day one plate) and incubated for 24 hours to allow the cells to attach to the bottom of the wells. Determination of viable cells was performed by adding 3-(4,5-dimethyl-2-thiazol)-2,5-diphenyl-2H-tetrazolium bromide (MTT; Sigma-Aldrich) to a final concentration of 0.5 mg/ml. After 3 hours incubation at 37°C and 5% CO_2_ in a humidified incubator, the formazan crystals were resolved by 10% SDS in 10 mM HCl and the absorption was measured at 560 nm. MTT measurements were performed daily for 5 consecutive days, as triplicates in three independent experiments. The absorption data were used to calculate the doubling time of each cell line (V. Roth 2006; wwww.doubling-time.com).

### Colony formation assay

Anchorage-independent cell proliferation was analyzed by soft agar colony formation assay. HCT116-LUC, HCT116-LUC-shNC and HCT116-LUC-shS100A4 cells were resuspended in RPMI-1640, supplemented with 10% FCS and 0.33% (w/v) agarose (Life Sciences Corporation). One × 10^3^ cells/ml were seeded into soft agar and colony growth was analyzed after 7 days by light microscopy. Only colonies with more than three cells were counted. Independent colony formation experiments were repeated twice, each in triplicate.

### Boyden chamber transwell migration and invasion assay

The cell lines HCT116-LUC, SW620, and DLD-1 and the respective clones expressing control- or S100A4-shRNA were used in cell migration and invasion analyses performed with Boyden chamber assay. 2.5 × 10^5^ cells were seeded into each transwell chamber with filter membranes of 12 μm pore size (Millipore, Germany). For invasion, filter membranes were coated with Matrigel (BD Biosciences; diluted 1:3 in growth medium) 4 hours before cell seeding, and fresh medium was added to the bottom chamber. After 24 hours (migration) or 72 hours (invasion), respectively, insets were removed and cells, which had migrated through the membrane to the lower chamber, were trypsinized and counted in a Neubauer chamber (LO-Laboroptik, Bad Homburg, Germany). Each well was counted ten times. Each migration or invasion experiment was performed in duplicate. The average number of migrated or invaded cells was determined from at least three independent experiments.

### Monitoring plasmid concentration in mouse blood after tail vein injection

Plasmid concentration in the blood of NOD/SCID mice after single tail vein injection of 10 μg SureSilencing™ shRNA expression plasmid (QIAGEN, Germany), supplemented with 2 μg aurintricarbolic acid (Sigma, Germany) in 200 μl sterile PBS, were determined by total DNA extraction of 200 μl blood samples (Quick Blood DNA Extraction Kit; Roboklon, Germany), taken before single tail vein injection and after 5 minutes, 30 minutes, 1, 2, 4, and 24 hours. Plasmid copy numbers were determined by SYBR Green based quantitative real time PCR (forward-primer 5´-agacaatcggctgctctgat-3´, reverse-primer 5´-caatagcagccagtcccttc-3´, 203 bp amplicon).

### Metastasis formation in xenografted mice

Animal experiments were performed in accordance with the UKCCCR guidelines and approved by the responsible local authorities (State Office of Health and Social Affairs, Berlin, Germany). For analysis of stably transfected shRNA-expressing cell lines, 3 × 10^6^ cells HCT116-LUC-shNC or HCT116-LUC-shS100A4 were transplanted at day zero into the spleens of 6 to 8 weeks old female NOD/SCID mice (randomly assigned to 8 mice per group). The mice were imaged twice a week in a NightOWL LB 981 system (Berthold Technologies, Germany) by intraperitoneal injection of 150 mg/kg D-luciferin (Biosynth, Switzerland) under temporal anesthesia with 35 mg/kg hypnomidate (Jassen-Cilag, Germany). Mice were sacrificed at day 28. Spleens (site of tumor injection) and livers (metastasis target organ) were removed and shock-frozen in liquid nitrogen. To evaluate systemic application of shRNA-expressing plasmids, 3 × 10^6^ HCT116-LUC cells were transplanted at day zero into the spleens of 6 to 8 weeks old female NOD/SCID mice (randomly assigned to 8 mice per group). Ten μg SureSilencing™ shRNA expression plasmid (SABiosciences), containing either S100A4-specific (shS100A4) or negative control (shNC) shRNA sequences, respectively, supplemented with 2 μg aurintricarbolic acid in 200 μl sterile PBS, were injected within 1 second into the tail vein at days 1, 5, 7, 9, 12, 14, 16, 19, 21, 23, and 26. The mice were imaged twice a week, as described above. Mice were sacrificed at day 27. Spleens and livers were removed and shock-frozen in liquid nitrogen. The level of liver metastasis was evaluated by scoring. The score for each liver was calculated as the sum of the volumes V of the individual metastases, calculated by V=L^2^ × W (with L as the length of the smaller axis, and W as the length of the larger axis). Luminescence signals of isolated organs were quantified with ImageJ. Serial consecutive cryosections were made from spleen tumors and liver metastases for immunohistochemistry or subsequent DNA and RNA isolations.

### Statistical analysis

Statistical analyses were performed with Sigma Stat Version 3.5. Depending on the normal distribution of the dataset, comparison of two groups was done either by Student's t-test or Mann-Whitney-Rank-Sum-Test. Comparison of more than two groups was performed by one-way analysis of variance (ANOVA) and Bonferroni post hoc multiple comparison. *P* values less than 0.05 were defined as statistically significant.

## Supplementary Figures


