# Supplementary material for: Systemic shRNA mediated knock-down of S100A4 in colorectal cancer xenografted mice reduces metastasis formation
Source: Oncotarget. 2012 Aug 7;3(8):783–97. doi: 10.18632/oncotarget.572 (PMC3478456; doi:10.18632/oncotarget.572)
Supplement: Supplementary file 1 [file oncotarget-08-783-s001.pdf]

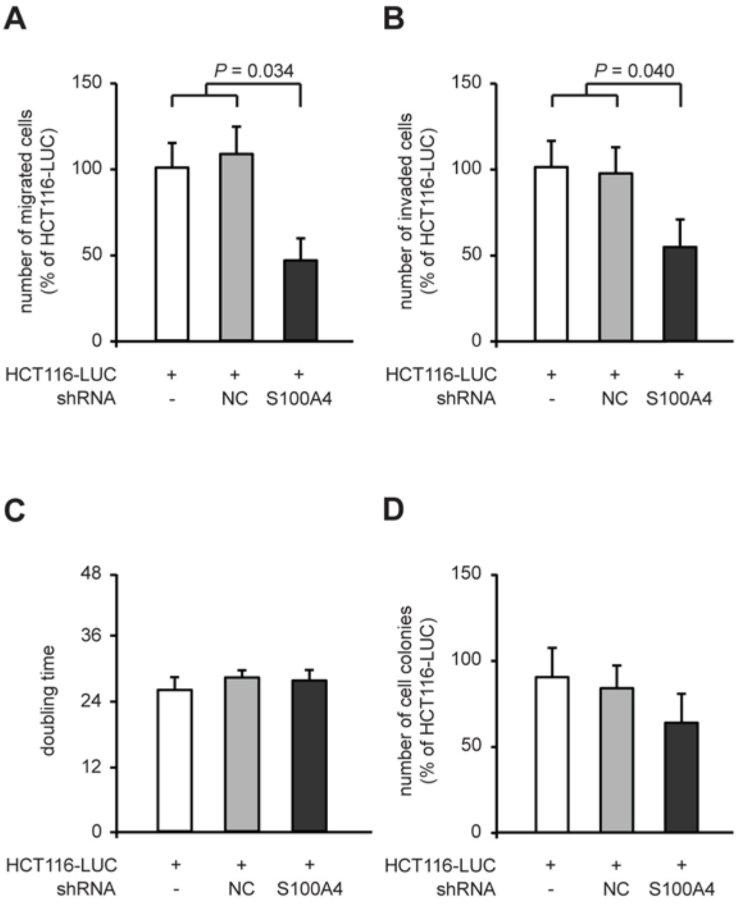

Supplemental Figure S1: S100A4 knock-down in HCT116-LUC cells reduces cellular migration and invasion, but has no effect on cellular growth

(A,B) Boyden Chamber assays of cell migration and invasion, respectively. Data represent the number of migrated or invaded cells normalized to the cell line HCT116-LUC as mean (n = 3) ± SD. (C) Doubling times of the cell lines HCT116-LUC, HCT116-LUC-shNC and HCT116-LUC-shS100A4 cells were determined by real time measuring of impedance based cell proliferation signals. Data represent mean (n = 3) ± SD. (D) Anchorage independent growth was assayed as the ability of HCT116-LUC, HCT116-LUC-shNC, and HCT116-LUC-shS100A4 cells to form colonies in soft agar. Colonies larger than three cells were counted and the results were normalized to HCT116-LUC cells. Data represent mean (n = 3) ± SD.
